# Iatrogenic sciatic nerve injury during liposuction and fat tissue grafting: a preventable surgical complication with devastating patient outcomes

**DOI:** 10.1186/s13037-020-00265-3

**Published:** 2020-10-23

**Authors:** Ibrahim E. Abdallah, Rita Ayoub, Raja Sawaya, Salim C. Saba

**Affiliations:** 1 Faculty of Medicine, American University of Beirut, P.O. Box 11-0236, Riad El-Solh, Beirut, 1107 2020 Lebanon; 2 Department of Surgery, American University of Beirut Medical Center, P.O. Box 11-0236, Riad El-Solh, Beirut, 1107 2020 Lebanon; 3 Department of Neurology, American University of Beirut Medical Center, P.O. Box 11-0236, Riad El-Solh, Beirut, 1107 2020 Lebanon

## Abstract

**Background:**

Liposuction and autologous fat transplantation represent widely used techniques in plastic surgery to correct or enhance contour irregularities in the face and body. While these techniques are assumed to be safe, liposuction and fat grafting impose a hidden risk for serious preventable surgical complications and adverse patient outcomes. We hereby report two cases of iatrogenic sciatic nerve injury and provide recommendations on how to prevent this serious surgical complication.

**Case presentation:**

We present two cases of sciatic nerve injury - one related to liposuction and the other related to gluteal lipo-augmentation. The first case is a 20-year-old female who presented to our institution with right leg weakness one week after undergoing scar revision and fat grafting in the left peri-oral region to correct peri-oral cicatricial banding and tissue atrophy. Fat was harvested from the medial thigh using a 3-mm cannula with low-pressure manual suction, utilizing minimal tumescent solution. Nerve conduction velocity and electromyography testing suggested a right-sided sciatic nerve lesion as a result of direct trauma. The patient was observed for a period of 4 months during which time she underwent physical therapy. At four months post-operatively, she had recovered completely. The second case is that of a 39-year-old female who presented to our institution with left-sided weakness of foot eversion and dorsiflexion five days after she had undergone liposuction of the thighs, flanks, and abdomen in addition to gluteal lipo-augmentation at an outside facility. The patient had undergone super wet liposuction in the areas of the abdomen, flanks and thighs. 200 mL of collected fat was then transplanted into each buttock at multiple levels. Post-operative computed tomography and electroneurography revealed acute left sided sciatic injury consistent with direct trauma to or compression of the sciatic nerve. The patient underwent an extensive regimen of physical therapy. Three months post-operatively, the patient had regained some motor function, but was lost to follow-up thereafter.

**Conclusion:**

The sciatic nerve is relatively superficial and vulnerable to injury in the upper thigh and lower buttock regions. Therefore, extreme care should be taken when working in these areas to avoid direct or indirect injury to the sciatic nerve by compression or traction.

## Background

Autologous fat transplantation is a very popular technique used by plastic surgeons to correct soft tissue defects. Procedures that employ fat transplantation include breast augmentation, scar and burn treatments, treatment of atrophies of the face and limbs, facial rejuvenation and gluteal augmentation [1]. This technique is carried out in three steps: liposuction, fat processing, and fat grafting [2]. Similar to other procedures, liposuction and fat grafting are associated with several complications. Liposuction complications include swelling, seroma, hematoma, skin necrosis, infection, skin surface irregularities, fat embolism, and thromboembolism, while those of fat grafting include fat necrosis, oil cyst formation, infection, skin irregularities, and embolism [3, 4]. While not as prevalent, nerve injuries have been reported following liposuction and/or fat grafting procedures [5, 6]. These injuries are the result of direct trauma, compression, or traction. One of the reasons of nerve traction and/or compression is the poor positioning of the patient during surgery, while direct trauma to the nerve is caused by the use of certain tools, such as cannulas, or the application of ultrasound or thermal energy, and may result in laceration or damage of small subcutaneous nerves [7, 8]. While infrequent, injury to the sciatic nerve during liposuction and/or fat grafting has been reported. This injury is under-reported, and those available are limited to cases of gluteal augmentation [5, 9]. In this report, we present two cases of sciatic neuropraxia that presented to our clinic – one following liposuction in the posterior thigh, and another following lipo-augmentation to the buttocks. We review the literature to gain a better understanding of the incidence of this unfortunate complication, and to make safety recommendations to avoid sciatic nerve injury in these procedures that are increasingly marketed as minimally invasive out-patient operations.

## Case presentation

### Overview

We present two patients who presented to our clinic in the American University of Beirut with apparent Sciatic Nerve dysfunction. These reports are based on patient accounts and operative records.

#### Case 1

A 20-year-old female, with a body mass index (BMI) of 26.1, presented to her surgeon with a lip commissure cicatricial contracture resulting from an electrical burn injury sustained during infancy. At one year of age, she underwent lower lip reconstruction with an Abbe flap to replace the lateral lip element which included the commissure. Physical examination revealed microstomia and an asymmetric lip due to a hypodynamic orbicularis oris at the Abbe flap recipient site. She also had a 2-cm-wide transverse scar that extended from the left oral commissure outwardly onto to the cheek with soft tissue atrophy of the left infra-labial region. Scar revision of the involved peri-oral and mental areas was performed. The atrophic chin area was grafted with fat harvested from the right medial thigh as the patient was thin.

The operation was performed under general anesthesia. The patient was placed in a frog-leg position for easy access to the medial thigh. The target area was infiltrated with a tumescent solution consisting of 40 mL of 0.5% xylocaine with 1:200,000 epinephrine. A 15-cm, 3-mm Mercedes liposuction cannula was used to harvest 50 mL of lipoaspirate using low-pressure manual suction. The cannula was passed through the postero-medial aspect of the thigh while pinching the areas being harvested to avoid passage deep to the subcutaneous layer. The fat was decanted by gravity and injected in standard fashion in the perioral region.

Three days post-operatively, the patient’s facial asymmetry was improved, but she complained of a foot drop on the side of the harvested thigh. She presented to our institution where examination revealed that she had severe weakness in the lateral compartment muscles of the leg, with anesthesia over the distribution of the common peroneal nerve. The patient was referred to the neurology department where a nerve conduction velocity (NCV) and electromyography (EMG) testing were conducted. Fractionated motor neurography results of the right peroneal nerve revealed low motor amplitudes and long durations across the different selected stimulation sites (Table 1). In addition, EMG results showed a lack of motor units and an absence of spontaneous activity in the right tibialis anterior and the right peroneus longus. Finally, EMG of the right tibialis posterior showed only a pair of motor units which had large amplitudes and durations. These results suggested a right-sided sciatic nerve lesion, proximal to the popliteal bifurcation, which involved predominantly the superior motor axons that give rise to the peroneal nerve.

**Table 1:** Fractionated motor neurography results of the peroneal nerves of the patient in case 1

Right Peroneal Nerve				Left Peroneal Nerve			
Recording site: Extensor digitorum brevis				Recording site: Extensor digitorum brevis			
**Stimulus site**	**Latency (ms)**	**Duration (ms)**	**Amplitude (mV)**	**Stimulus site**	**Latency (ms)**	**Duration (ms)**	**Amplitude (mV)**
Ankle	3.5	8.5	2.2	Ankle	3.2	6.2	7.0
Fibula (head)	11.0	9.3	2.0	Fibula (head)	9.2	6.4	6.5
Popliteal fossa	12.6	9.4	2.4	Popliteal fossa	10.9	6.5	6.7

For both nerves, the extensor digitorum brevis was selected as the recording site, and the latency (ms), duration (ms) and amplitude (mV) were measured across three different stimulus sites. Note the decreased amplitudes and increased durations of the wave forms on the right Peroneal Nerve

The patient underwent an aggressive physical therapy regimen that involved daily muscle ranging exercises along with electrical stimulation. Additionally, she underwent targeted mirror box therapy. Six weeks post-operatively, the patient began to show marked improvements in foot dorsiflexion and eversion. An additional movie file shows this in more detail (see Additional file 1). By 4 months post-operatively, the patient had regained normal gait and function.


**Additional file 1.** Patient of case 1 undergoing mirror box therapy. This video depicts the patient of case 1, almost 3 months following surgery, undergoing physical therapy utilizing mirror box therapy. While the effectiveness of this method is still under investigation in peripheral motor nerve injury, it is evident that the patient is able to dorsiflex the affected foot, implying improved function in the motor division of the Sciatic Nerve.

#### Case 2

A 39-year-old female, with a BMI of 28, who underwent liposuction of the thighs, flanks, and abdomen in addition to gluteal lipo-augmentation at an outside facility, presented 5 days post-operatively to our institution with left-sided weakness of foot eversion and dorsiflexion consistent with injury along the peroneal nerve axis. Other outstanding findings included a hemoglobin level of 7.9 mg/dL, although the patient was hemodynamically stable and otherwise asymptomatic. Operative records revealed that the patient underwent super wet liposuction with infiltration of a total of 2 L of standard Kline solution into the abdomen, flanks and thighs. 1.8 L of lipoaspirate, was obtained from the patient in the supine position. The patient was then placed in the prone position, and 200 mL of decanted fat was then transplanted into each buttock at multiple levels. Total surgical time was 2.5 h, and the patient had no complaints in the immediate post-operative period.

**Fig. 1:**
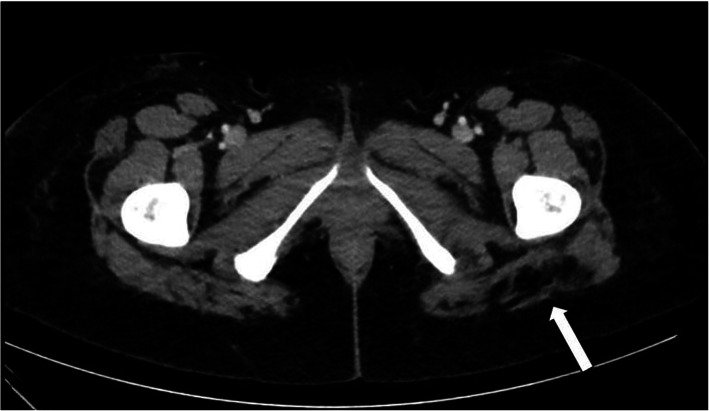
Computed tomography (CT) image of the lower gluteal region of the patient in case 2. White-colored areas correspond to the bones. The white arrow indicates areas of low attenuation (black) corresponding to significant fat within the inferior aspect of the left Gluteus Maximus muscle adjacent to the sciatic nerve (SN)

**Fig. 2:**
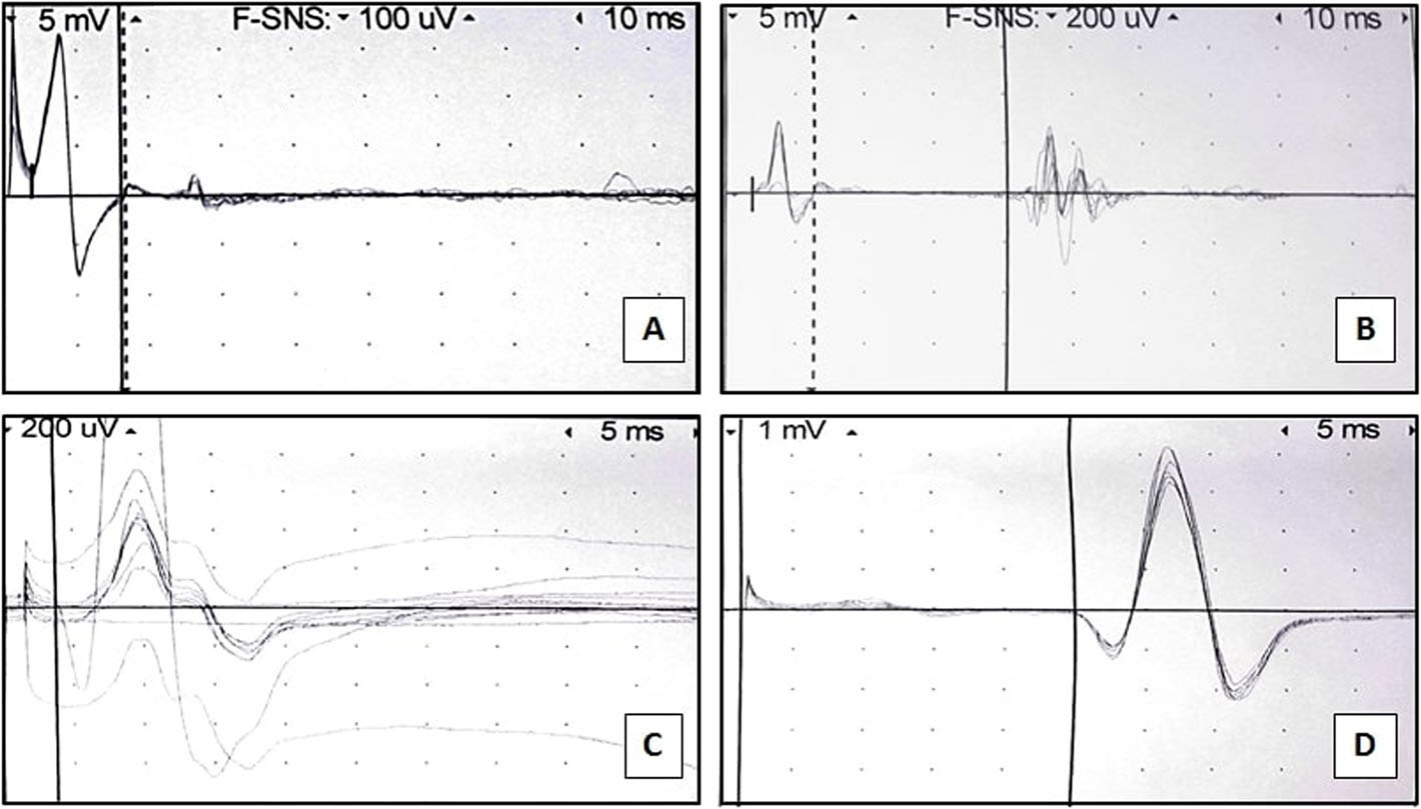
F-waves (**a**, **b**) and H-reflexes (**c**, **d**) of the left and right posterior Tibial nerves. F-waves correspond to the left posterior Tibial nerve (**a**) and right posterior Tibial nerve (**b**) and indicate absence of transmission in the distal motor nerve on the left. H-reflexes correspond to the left posterior Tibial nerve (**c**) and right posterior Tibial nerve (**d**) and indicate an absent reflex between type IA afferent and efferent nerves of the same muscle on the left. F-waves and H-reflexes corresponding to the right posterior Tibial nerve are normal

Post-operative computed tomography (CT) in our institution revealed distension of the inferior aspect of the left Gluteus Maximus muscle with fat in an area adjacent to the sciatic nerve (Fig. 1). The involved aspect of the left Gluteus Maximus was 1.7 times the thickness of the right side. The remainder of the study revealed edema in the remaining areas of the posterior thighs and abdomen consistent with standard post-liposuction changes. NCV studies of the lower extremities indicated the absence of posterior Tibial F-responses and posterior Tibial H-reflex on the left side compared to normal responses on the right side (Fig. 2). Based on these findings, it was concluded that the patient suffered from an acute left sided sciatic nerve lesion above the bifurcation in the area of the buttocks.

The patient underwent an extensive regimen of physical therapy that consisted of passive range of motion exercises. Three months post-operatively, the patient had regained some motor function, but was lost to follow-up thereafter.

## Discussion

Several reported neuropathies in cosmetic procedures resulted from pre-existing conditions that the patient had at the time of the surgery, rather than a direct injury to the nerve in question. One report of vision loss following liposuction was attributed to the patient’s idiopathic intracranial hypertension [10]. In another study, perioperative anemia and hypotension were major contributors to vision loss following liposuction [11]. Other studies cited injury to the sensory peripheral nerves of the lower abdomen and thigh. However, the majority of these reports involved small unnamed superficial sensory nerves. We report for the first time, two cases of traumatic sciatic nerve injury sustained during liposuction or fat grafting in the upper thigh and lower gluteal region. Although we initially suspected a positional injury to the Common Peroneal Nerve [12], electroneurographic studies demonstrated injury to the peroneal division of the sciatic nerve in the mid-thigh. The lateral and more superficial orientation of the peroneal division in the thigh renders it more susceptible to blunt trauma during liposuction.

Extensive review of the literature revealed only two reports of sciatic nerve injury during cosmetic procedures. Rawlani et al. reported a patient who underwent combined abdominoplasty and mastopexy surgery in the Fowler position that did not exceed 3 hours. Evidence of proximal compression of the sciatic nerve was seen in the form of a stage I pressure ulcer in the lower buttock region [13]. Kiermeir et al. reported sciatic nerve injury in two massive weight loss patients who underwent combined abdominoplasty and thigh lift surgery in the semi-recumbent positions [14]. Both patients regained full function within 6 months. In contrast to these reports which were related to sciatic nerve compression and stretch injuries from positioning, our cases are suggestive of direct blunt trauma to the sciatic nerve. Moreover, the patient in case 1 spent only 30 min in the frog leg position and was at no point in hip flexion. The patient in case 2 spent no more than 1.5 h each in the supine or prone positions, with only mild hip flexion in the latter. The literature cites sciatic nerve injury related to poor positioning from long vertebral surgeries, in which the patient is in excessive hip flexion. However, no references were found that reported sciatic nerve injury related to surgical positioning in short-duration procedures.

Scenarios similar to our case 2 have only been reported by Cardenas-Mejia et al. and Vasilakis et al. as recently as 2009 and 2018, respectively. The former reported a bilateral sciatic nerve axonotmesis in a patient after grafting only 200 cc of fat in each buttock, with most of the volume injected in the Gluteal muscles [15]. Vasilakis also reported on a bilateral case of sciatic neuropathy, following gluteal augmentation, with only partial recovery on one side after more than one year of physical therapy [5]. In both cases, electroneurography and imaging revealed direct compression and/or trauma to the sciatic nerve. While the second patient in our report was showing signs of clinical improvement, CT imaging revealed likely compression, and possibly direct trauma, as evidenced by fat distension adjacent to the sciatic nerve sheath (Fig. 1).

Many cases of sciatic nerve injury are reported in relation to hip arthroplasty. This occurs due to the anatomic vulnerability of the sciatic nerve as it exits the greater sciatic foramen and lies superficial to the Gemelli, Obturator internus and Quadratus femoris muscles. In the sub-piriformis area, the sciatic nerve runs in the ischio-trochanteric channel where it is most susceptible to injury at the sciatic cutaneous projection [16, 17]. In patients of normal BMI, we believe that the sciatic nerve is relatively superficial and thus, vulnerable to injury in the lower buttock and upper thigh region. Mechanism of injury was likely a combination of direct trauma (case 1) and possibly direct trauma combined with compression (case 2), and they underscore the need to avoid the upper posterior and lower gluteal regions when performing either liposuction or fat grafting. These cases are likely under-reported and should serve as cautionary examples to all surgeons who work in the lower buttock and upper thigh regions.

### Safety recommendations

**Fig. 3:**
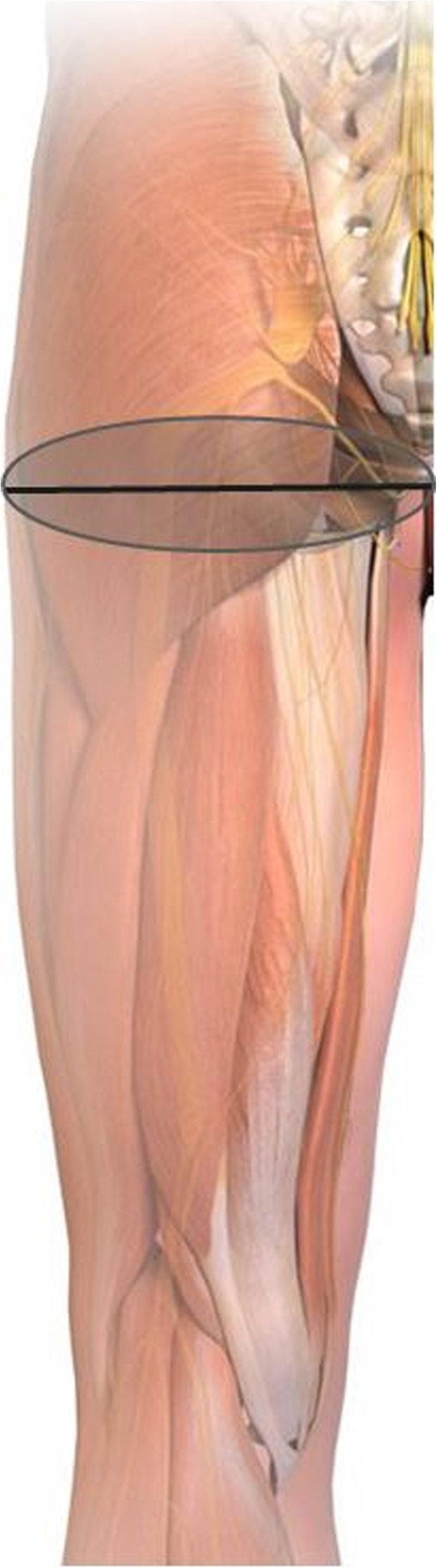
Danger zone in the upper thigh and lower gluteal region corresponding to the ischio-trochanteric channel. The zone (light gray zone) centers on an imaginary line (represented by a black line) that crosses between the coccyx and the greater trochanter of the femur. In hip flexion, the sciatic nerve (SN) may become more superficial, as the overlying muscles are stretched, and thus prone to blunt trauma

Several measures will be outlined in order to reduce sciatic nerve injury when performing liposuction or fat grafting in the thigh and buttock region. Avoiding liposuction in the upper posterior thigh or lower buttock regions is of utmost importance, especially in patients of normal or low BMI. In cases where liposuction of the medial thigh is done, extreme care to ensure subcutaneous-only passage is to be taken to avoid inadvertently trespassing onto the lower buttock and upper thigh danger zones, as these areas overlie the ischio-trochanteric channel where the sciatic nerve becomes relatively superficial as it exits the sub-piriformis channel (Fig. 3). As an additional and final precaution, fat transplantation in the buttocks must only be performed in the subcutaneous plane, away from the Gluteus muscles. This stems from a recent recommendation made by the Safety in Fat Grafting Task Force when the American Society of Plastic Surgeons cited a mortality rate of 1 for every 3000 gluteal lipo-augmentation procedures. In all cases, fatal fat emboli were identified as the cause, giving gluteal lipo-augmentation the highest mortality of any cosmetic procedure [18].

## Conclusion

Suction-assisted lipectomy and gluteal lipo-augmentation remain among the most frequently performed aesthetic procedures in the United States and worldwide. Peripheral nerve injuries are not an uncommon occurrence as these procedures continue to gain more widespread use. We believe that iatrogenic sciatic nerve injury related to liposuction in the thigh region, and/or gluteal lipo-augmentation, is under-reported and should be included in the pre-operative discussion and consent of all surgical candidates. It is crucial that this type of complication be reported so that coherent guidelines for patient selection and safety measures are ensured.

## Data Availability

Not Applicable.

## Abbreviations

BMI: Body mass index
CT: Computed tomography
EMG: Electromyography
NCV: Nerve conduction velocity
